# SHOULDER PAIN, ULTRASOUND CHANGES, AND FUNCTIONAL PERFORMANCE IN OBESE PATIENTS

**DOI:** 10.1590/1413-785220243206e282000

**Published:** 2025-01-10

**Authors:** Fabiana Alves Costa Menegassi, Glaucus Cajaty Martins, Zartur José Barcelos Menegassi, Sérgio Augusto Lopes de Souza, Bianca Gutfilen, Fábio Ramos Costa

**Affiliations:** 1. Instituto Nacional de Traumatologia e Ortopedia, Rio de Janeiro, RJ, Brazil; 2. Hospital Federal de Ipanema, Rio de Janeiro, RJ, Brazil; 3. Departamento de Ortopedia e Traumatologia, Faculdade de Medicina, Universidade Federal do Rio de Janeiro, Rio de Janeiro, RJ, Brazil; 4. Departamento de Radiologia, Faculdade de Medicina, Universidade Federal do Rio de Janeiro, Rio de Janeiro, RJ, Brazil; 5. Departamento de Ortopedia, FC Clínica de Traumatologia Esportiva, Salvador, BA, Brazil

**Keywords:** Shoulder, Obesity, Pain, Diagnosis, Ombro, Obesidade, Dor, Diagnóstico

## Abstract

Objective: To evaluate the prevalence of shoulder pain, level of functional performance, and morphological involvement of the rotator cuff on ultrasound in morbidly obese patients. Methods: The study included 54 morbidly obese patients receiving follow-up care in a bariatric surgery outpatient clinic, which were compared with a control group consisting of 49 participants. Presence of shoulder pain, shoulder functional performance, ultrasound of the rotator cuff and blood tests were the parameters evaluated. Results: The obese group showed a greater incidence of shoulder pain (p < 0.0001; OR: 17.5), lower functional performance according to UCLA (p < 0.0001; OR: 7.5) and DASH scales (p < 0.0001) and a greater incidence of pathological ultrasound and c-reactive protein test changes (p < 0.0001). Conclusions: These findings suggest that being overweight is an important exacerbating factor for shoulder pain, lower shoulder functional performance and pathological changes of the rotator cuff and c-reactive protein test. *Level of Evidence II, Cross-sectional study.*

## RESUMO

Objetivo: Avaliar, em pacientes com obesidade mórbida, a prevalência de dor no ombro, o nível de desempenho funcional e o comprometimento morfológico do manguito rotador na ultrassonografia. Métodos: Foram incluídos 54 pacientes com obesidade mórbida, acompanhados em um ambulatório de cirurgia bariátrica, comparados com um grupo controle de 49 indivíduos. Foram avaliados os seguintes parâmetros: presença de dor no ombro, desempenho funcional do ombro, imagens ultrassonográficas do manguito rotador e exames de sangue. Resultados: O grupo obeso apresentou maior incidência de dor no ombro (p < 0,0001; OR: 17,5), menor desempenho funcional segundo as escalas UCLA (p < 0,0001; OR: 7,5) e DASH (p < 0,0001), maior incidência de alterações patológicas na ultrassonografia e aumento da proteína c-reativa (p < 0,0001). Conclusões: Estes dados sugerem que o excesso de peso é importante fator de exacerbação da dor no ombro, menor desempenho funcional do ombro, alterações patológicas do manguito rotador e aumento da proteína c-reativa. *Nível de Evidência II, Estudo transversal controlado.*

## INTRODUCTION

 According to the World Health Organization (WHO), obesity is a condition characterized by a percentage of body fat large enough to impair one’s health, ^1^ resulting from a complex interaction of variables including genetics, lifestyle, dietary habits, caloric expenditure, nutritional factors, and adipocyte metabolism. ^2^ ^,^ ^3^ Recent research reports an association between obesity and greater pain or osteoarthritis (OA) in non-load bearing or traction joints, including the shoulder joint, suggesting possible association of other factors such as low-level systemic inflammation ^4^ ^-^ ^6^ and genetic factors. ^7^

 This chronic inflammation process is directly related to adipokines, bioactive peptides originating from visceral fat that have roles in insulin action and in inflammatory cytokines production in chondrocytes. ^4^ ^,^ ^6^ In morbidly obese patients (BMI ≥ 40 kg/m2), the prevalence of shoulder pain, level of functional performance, and morphological involvement of the rotator cuff on ultrasound have yet to be adequately described. Determining clinical and imaging parameters that could point to the need for early intervention are important when formulating therapeutic and preventive strategies. ^8^ The primary objective of this study was to assess the prevalence and intensity of shoulder pain and functional performance in morbidly obese patients selected for bariatric surgery. Secondary objectives included assessing the prevalence of morphological involvement of the rotator cuff on ultrasound and analyzing blood tests, including lipidemia, glycemia, and inflammatory markers, comparing their results with those of a control group. 

## METHODS

### Study design

 This was a controlled cross-sectional study conducted in accordance with the norms set forth by the World Medical Association’s Declaration of Helsinki and approved by the Research Ethics Committee of Ipanema Hospital, Rio de Janeiro, Brazil, under Certificate of Presentation for Ethical Appraisal (CAAE) No. 69073215.2.0000.5646, opinion No. 2.127.775. Data were collected between March 2020 and April 2021 in Rio de Janeiro, Brazil. All study participants signed an informed consent form. The study sample comprised 54 morbidly obese patients, 8 (14.8%) men and 46 (85.2%) women, who were selected for bariatric surgery for weight reduction or diabetes control. Additionally, 49 healthcare professionals in active work—6 men (12.2%) and 43 women (87.8%)—were included in the control group. Patients aged ≥18 years and who provided consent to participate in the study were included. Individuals with fractures, those who had undergone previous surgery in the upper limbs, and those who presented rheumatologic diseases were excluded. Pains was assessed by the 0-10 Visual Analog Scale (VAS), in which 0 is painless and 10 is maximum pain. VAS score of >3 was considered strong pain and significant for odds ratio analysis. Functional evaluation of the shoulders was performed using the Disabilities of the Arm, Shoulder, and Hand (DASH) and University of California at Los Angeles (UCLA) functional scales. DASH ^9^ is a decreasing scale, i.e., a lower score indicates better functional performance, that assesses the functional performance of the upper limbs. A value of zero would mean the absence of any deficit, whereas a value of 150 would mean the worst possible impairment. The UCLA scale ^10^ assesses only the shoulders where the highest possible score is 35, with values below 20 indicating deficits in functional performance. Participants’ shoulders were morphologically evaluated via ultrasound imaging to verify impairment using the high frequency 3-12MHz linear transducer, Philips EPIQ 7 Ultrasound Machine, as per the European Society of Musculoskeletal Radiology guidelines of ultrasound technical for shoulder. All ultrasound shoulder examinations were performed by trained musculoskeletal sonographers. Erythrocyte sedimentation rate by the Westergren method, serum total cholesterol serum levels (low density lipoprotein and high-density lipoprotein fractions) and triglycerides by enzymatic-colorimetric method, C reactive protein (CRP) by conventional method, and glycated hemoglobin (HbA1c) by the high-performance liquid chromatography method were the laboratory parameters used for metabolic evaluation. 

### Statistical analyses

 Data analysis focused on the limb with the most symptoms in each patient (i.e., the limb with the highest VAS score) to prevent bias in the statistical analysis as when both limbs (shoulders) are evaluated as statistically separate units, as previously described by Menz. ^11^ If a participant reported no pain or functional complaints relative to a specific upper limb, the shoulder on the dominant side was selected for evaluation. Descriptive analysis presented the observed data as tables, expressed as adequate measures of central tendency and dispersion for numeric data and as frequencies and percentages for categorical data. Intergroup comparisons regarding clinical and laboratory variables were performed using Student’s t-test for independent samples or Mann–Whitney U test for numeric variables, and the chi-squared test (χ2) or Fisher’s exact test for categorical variables. Association of the VAS, DASH, and UCLA scales with the laboratory variables was calculated using Spearman’s correlation coefficient. Non-parametric methods were used as all variables, except for age, showed non-normal distribution following rejection of the normality hypothesis by the Shapiro–Wilk test. Significance level was set at 5%. Statistical analysis was processed using the statistical software program SAS® System, version 6.11 (SAS Institute, Inc., Cary, North Carolina, USA). 

## RESULTS

Table 1 presents the values observed regarding the clinical and laboratory variables for each group (bariatric surgery candidates and control group) and the corresponding descriptive level (p-value) of the statistical test. Excepting age, the numeric variables evaluated did not follow a normal (Gaussian) distribution at 5% level, according to the Shapiro–Wilk test, in at least one of the groups. Thus, quartile measurements were most appropriate for data summarization—i.e., median, interquartile range (IQR) from the first to the third quartile (Q1–Q3), minimum value, and maximum value. Numeric data were expressed either as means and standard deviations or as medians and IQR (Q1–Q3), and compared using Student’s t-test for independent samples or the Mann–Whitney U test. Categorical data were expressed as frequencies (n) and percentages (%), and compared using either the chi-squared test (χ2) or Fisher’s exact test ( Table 2 ). 

**Table 1.** Numeric clinical and laboratory variables for each group.

Variable	Case group			Control group			pvalue
	n	Median	IQR	n	Median	IQR	
Clinical							
Age (years)	54	48 ± 10		49	53 ± 11		0.021
VAS	54	7	4–8	48	0	0–0	< 0.0001
DASH	54	59	38–81	49	35	29–52	< 0.0001
UCLA	54	22	17–31	48	35	29–35	< 0.0001
BMI (kg/m2)	54	41	39–45	49	27	25–29	< 0.0001
Laboratory							
ESR	15	24	17–40	20	21	15–32	0.50
CRP	32	0.87	0.43–1.49	27	0.23	0.12–0.36	< 0.0001
HbA1c	38	5.70	5.40–6.00	40	5.55	5.30–5.95	0.29
Total cholesterol	41	197	166–236	40	196	173–230	0.76
Triglycerides	40	108	79–169	40	95	64–162	0.49
HDL	40	47	41–56	40	50.5	41–68	0.73
LDL	40	117	97–136	40	116	98–137	0.80

IQR: interquartile range (quartiles 1–3); VAS: Visual Analog Scale; DASH: Disabilities of the Arm, Shoulder, and Hand scale; UCLA: University of California at Los Angeles scale; BMI: body mass index; ESR: erythrocyte sedimentation rate; CRP: C-reactive protein; HbA1c: glycated hemoglobin; HDL: high-density lipoprotein cholesterol; LDL: low-density lipoprotein cholesterol. Age was expressed as mean ± standard deviation and compared using Student’s t-test for independent samples. The other variables were expressed as medians and IQR and compared using the Mann–Whitney U test.

**Table 2.** Categorical clinical variables for each group.

Variable	Case group			Control group			pvalue
	N	%		N	%		
Gender							
Male	8	14.8		6	12.2		0.70
Female	46	85.2		43	87.8		
Ultrasound changes							
Yes	42	84.0		15	31.9		< 0.0001
No	8	16.0		32	68.1		
Ultrasound findings							
Normal	8	16.0		32	68.0		< 0.0001
Tendinosis	30	60.0		13	27.7		
Tendinosis + rupture	12	24.0		2	4.3		
Side with greater pain							
Neither	7	13.5		36	75.0		< 0.0001
Right	27	51.9		8	16.7		
Left	18	34.6		4	8.3		
Dominant side							
Right	48	92.3		46	93.9		0.53
Left	4	7.7		3	6.1		
VAS > 3							
Yes	42	77.8		8	16.7		< 0.0001
No	12	22.2		40	83.3		
UCLA < 20							
Yes	18	33.3		3	6.2		0.001
No	36	66.7		45	93.8		

VAS: Visual Analog Scale; DASH: Disabilities of the Arm, Shoulder, and Hand scale; UCLA: University of California at Los Angeles scale. Data were expressed as frequencies (n) and percentages (%) and compared using either the chi-squared test (χ2) or Fisher’s exact test.

 The case group showed a significantly lower age (p = 0.021) and UCLA score (p < 0.0001), but a significantly higher VAS (p < 0.0001), DASH (p < 0.0001), BMI (p < 0.0001), and CRP levels (p < 0.0001) than the control group. We identified no significant intergroup difference for the other numeric variables ( Table 1 ). Regarding categorical variables, the obesity group showed significantly higher percentages of ultrasound shoulder examination changes (p < 0.0001), tendinosis-type changes and association with rupture (p < 0.0001), right side with more pain compared to the left side (p < 0.0001), VAS > 3 (p < 0.0001), and UCLA < 20 (p = 0.001) than the control group. We found no significant intergroup difference for the other categorical variables ( Table 2 ). Odds ratio (OR) in the obesity group was 17.5 for VAS > 3 (95% confidence interval [CI]). In the subgroup with ultrasound shoulder changes, only CRP levels appeared to be significantly higher than in the subgroup with normal ultrasound examination (p = 0.008). We identified no significant intergroup difference for the other laboratory variables ( Table 3 ). 

**Table 3.** Laboratory variables according to ultrasound findings.

Variable	Ultrasound with changes			Normal ultrasound			p value
	n	Median	IQR	N	Median	IQR	
Laboratory							
ESR	24	18	15–29.5	11	23	21–41	0.21
CRP	40	0.75	0.303–1.46	19	0.23	0.14–0.52	0.008
HbA1c	46	5.70	5.30–6.05	32	5.50	5.40–5.93	0.23
Total cholesterol	48	198	163–238	33	192	178–230	0.97
Triglycerides	48	116.5	68–169	32	98.5	66–162	0.59
HDL	48	49	39–61	32	50	43–66	0.88
LDL	48	115	97–135	32	118	98–143	0.57

IQR: interquartile range (quartiles 1–3); ESR: erythrocyte sedimentation rate; CRP: Creactive protein; HbA1c: glycated hemoglobin; HDL: high-density lipoprotein cholesterol; LDL: low-density lipoprotein cholesterol. DU+0078ds2ata were expressed as medians and IQR and compared using the Mann–Whitney test.

Table 4 shows a weak direct correlation between VAS score and HbA1C levels (r = 0.263; p = 0.020; n = 78). Thus, a higher HbA1C level was associated with a higher VAS score. Another weak correlation was observed between the DASH score and CRP levels (r = 0.269; p = 0.039; n = 59), suggesting that a higher CRP level was associated with a higher expected DASH score. A weak inverse correlation was found between the UCLA score and CRP levels (r = −0.334; p = 0.010; n = 59), indicating that the higher the CRP level, the lower the UCLA score. 

**Table 4.** Correlation between the VAS, DASH, and UCLA scales and the laboratory variables in the entire sample

Variable	VAS			DASH			UCLA		
	N	rs	pvalue	N	rs	pvalue	n	rs	pvalue
ESR	35	−0.108	0.54	35	0.018	0.92	35	0.012	0.94
CRP	59	0.226	0.085	59	0.269	0.039	59	−0.334	0.010
HbA1c	78	0.263	0.020	78	0.168	0.14	78	−0.146	0.20
Total cholesterol	81	0.003	0.98	81	0.048	0.67	81	−0.033	0.77
Triglycerides	80	0.008	0.94	80	0.213	0.058	80	−0.115	0.31
HDL	80	0.126	0.26	80	0.062	0.59	80	−0.030	0.79
LDL	80	−0.025	0.83	80	−0.013	0.91	80	0.008	0.94

VAS: Visual Analog Scale; DASH: Disabilities of the Arm, Shoulder, and Hand scale; UCLA: University of California at Los Angeles scale; rs: Spearman’s correlation coefficient; BMI: body mass index; ESR: erythrocyte sedimentation rate; CRP: Creactive protein; HbA1c: glycated hemoglobin; HDL: high-density lipoprotein cholesterol; LDL: low-density lipoprotein cholesterol.

 We observed a predominance of right-handed individuals among the study participants, with 92.3% in the obesity group and 93.9% in the control group. The right side presented pain in 51.9% of the obesity group participants, the left side was affected in 34.6%, and 13.5% had no pain. Conversely, these percentages were 16.7%, 8.3%, and 75%, respectively, in the control group ( Table 2 ). 

## DISCUSSION

 The analyzed outcomes revealed that morbidly obese patients had a higher incidence of shoulder pain, at much higher levels than the control group of active workers. Previous studies ^6^ conducted with industrial workers and in broader population groups have shown a correlation between obesity and painful shoulder joint impairment. Functional impairment evaluation revealed a significant functional shoulder limitation in the obese group. Studies that systematically evaluated shoulder performance in groups of obese individuals corroborate that lower shoulder functionality is typically observed in cases of significant obesity. ^6^ Eichinger et al. ^12^ proposed the causal hypotheses of the effect of arm weight, broader torso, deconditioning, or a combination of these factors as a reason for this limitation. In the present study, morphological evaluation of the rotator cuff using ultrasound revealed how often this anatomical structure was affected in morbidly obese individuals: 60% patients presented tendinosis and 24% had tendinosis and rupture of the rotator cuff, compared with 27.7% tendinosis and 4.3% partial cuff tear in the control group. These values are significantly higher in obese people than in the general population within the same age range, ^8^ with an expected percentage of rotator cuff tears (both partial and total) of 6.7% and 12.8%, respectively. Obesity has been listed as a risk factor for tendinopathy, and both mechanical stress and low-level inflammatory process associated with obesity have been considered etiological factors. ^4^ ^-^ ^6^ ^,^ ^13^ Chronic inflammatory processes caused by obesity predispose obese patients to other chronic diseases such as type-II diabetes mellitus, cardiovascular disorders, degenerative and autoimmune diseases—all of which are risk factors for musculoskeletal diseases. This would partially explain the high degree of musculoskeletal involvement observed in these patients; however, low-level inflammatory process must be considered a direct etiological factor associated with this involvement. ^4^ ^,^ ^6^ ^,^ ^8^ Despite efforts to attempt age pairing, patients in the control group were slightly older than those in the obesity group. Older age has a known correlation with worsened functional scores, increased shoulder pain, and increased rates of rotator cuff tears. ^14^ This consequently reinforced that functional impairment and morphological involvement of the rotator cuff were extremely significant in the obesity group, despite the younger mean age, showing significantly higher changes than the control group. Serum HbA1c levels, total cholesterol, and cholesterol fractions values were similar for both groups, possibly because the obese participants were awaiting bariatric surgery and therefore had undergone a rigorous program of clinical control and surgery preparation. This could explain why no significant differences were observed in these serum parameters in relation to the control group, which intends to reflect the general population. CRP level indirectly measures an in-progress inflammatory process. Although some laboratory parameters (glucose, cholesterol and fractions) could be controlled with diet and medical measures in the pre-surgery group, the end organs alterations were not reverted. Short-time period control of glucose levels does not alter morphological alterations in kidney vessels or retinopathy in diabetic patients. Similarly, control of these laboratory parameters does not influence the morphological changes and inflammatory process in knee joints, ankle joints and spine caused by morbid obesity ^8^ —which justifies the high CRP levels. Despite impossibility to establish a correlation between lipid and glycemic levels and rotator cuff tears, the requirement to be under good clinical control before undergoing surgery could explain the serum cholesterol levels and its fractions mostly tending to normal values in the obesity group. This does not rule out the possibility of these individuals having presented high cholesterol and HbA1c levels in the past, and that these factors might have led to a rotator cuff tear. A cross-sectional study showed a snapshot of the studied group at a specific moment. ^8^ CRP levels appeared to be significantly higher in the obesity group compared with the control group. This finding corroborates those of other articles, and demonstrate that obesity is an inflammatory process that adversely affects the joints, muscles, and tendon insertions. ^4^ ^-^ ^6^ ^,^ ^13^ ^,^ ^15^ Statistical analysis clearly showed the correlation between elevated serum CRP levels and tendinosis and partial and total rotator cuff tears. The statistical correlation found between increased CRP levels and poorer functional performance in the DASH and UCLA scales highlights the systemic nature of obesity as a metabolic disease ^13^ ^,^ ^14^ ^,^ ^16^ whose effects on the musculoskeletal system were mediated via humoral agents such as adipokines, free radicals, and interleukins. ^16^

### Limitations

Study limitations include its cross-sectional nature, which can provide correlations between the studied parameters but cannot determine a specific cause–effect relation between them. Additionally, there was no adequate pairing of participants’ ages. However, the poorer functional results observed in the group of younger obese patients further reinforce the significance of functional impairment in the obese population. Our study sample was relatively small, yet significant results were obtained which highlights the comprehensive extent of functional shoulder impairment and pain that the studied individuals suffer.

### What Does This Study Add To Current Knowledge?

Notably, the present study is an original research addressing a very specific study population composed of overweight individuals selected for bariatric surgery. Most studies in the literature refer to overweight individuals or to obesity defined generically as BMI ≥ 30 kg/m2, rarely analyzing subgroups with BMI ≥ 40 kg/m2. Data obtained in this study may be useful for understanding musculoskeletal system diseases resulting from metabolic syndrome-related aspects of overweight and obesity that affect the upper limbs, particularly the shoulders. These findings may be especially useful for formulating preventive and therapeutic strategies for this particular subgroup.

## CONCLUSIONS

Morbidly obese patients presented a higher incidence of shoulder pain, loss of function and pathological changes, as well as higher c-reactive protein than the control group.
